# Renal protection in diabetes: lessons from ONTARGET^®^

**DOI:** 10.1186/1475-2840-9-60

**Published:** 2010-10-01

**Authors:** Eberhard Ritz, Roland E Schmieder, Carol A Pollock

**Affiliations:** 1 Dept. Internal Medicine, Division Nephrology, Heidelberg, Germany; 2 Universitat Erlangen, Medizinische Klinik IV, Erlangen, Germany; 3 Dept. Medicine, Royal North Shore Hospital, St Leonards, Australia

## Abstract

Hypertension is an important independent risk factor for renal disease. If hypertension and chronic renal disease co-exist, as is common in patients with diabetes mellitus, the risk of cardiovascular disease is heightened. The importance of rigorous blood pressure control is recognized in current guidelines, with a recommended target of office blood pressure of < 130/80 mmHg; although ambulatory blood pressure may be more appropriate in order to identify the 24-hour hypertensive burden. Even lower blood pressure may further reduce the progression of chronic kidney disease, but the incidence of cardiovascular events may increase. Albuminuria not only indicates renal damage, but is also a powerful predictor of cardiovascular morbidity and mortality at least in patients with high cardiovascular risk and potentially pre-existing vascular damage. Management of the multiple factors for renal and cardiovascular disease is mandatory in the diabetic patient. The renin-angiotensin system (RAS) plays a pivotal role in the progression of renal disease, as well as in hypertension and target-organ damage. The use of agents that target the RAS confer renoprotection in addition to antihypertensive activity. There is extensive evidence of the renoprotective effect of angiotensin II receptor blockers (ARBs), and specifically telmisartan. In addition to providing 24-hour blood pressure control, clinical studies in patients with diabetes show that telmisartan improves renal endothelial function, prevents progression from microalbuminuria to macroalbuminuria, slows the decline in glomerular filtration rate and reduces proteinuria in overt nephropathy. These effects cannot be solely attributed to blood pressure control. In contrast to other members of the ARB class, the renoprotective effect of telmisartan is not confined to the management of diabetic nephropathy; slowing the progression of albuminuria has been demonstrated in the ONgoing Telmisartan Alone and in combination with Ramipril Global Endpoint Trial (ONTARGET^®^), which included diabetic and non-diabetic patients at high risk of cardiovascular events.

## Introduction - the ONTARGET^® ^study

The ONgoing Telmisartan Alone and in combination with Ramipril Global Endpoint Trial (ONTARGET^®^) was the largest ever study of an angiotensin II receptor blocker (ARB) [1]. It compared telmisartan with ramipril, and the combination, with a treatment duration of up to 5.5 years. ONTARGET^® ^was notable not only for the size of the patient population (over 25,000), but also for the broad inclusion criteria (patients could have coronary artery disease, peripheral arterial occlusive disease, cerebrovascular event or diabetes mellitus with end-organ damage). Patients were screened for tolerance to angiotensin-converting enzyme (ACE) inhibitors before trial entry.

ONTARGET^® ^showed that telmisartan was as effective as ramipril, the ACE inhibitor whose efficacy in reducing cardiovascular events was established in the Heart Outcomes Prevention Evaluation (HOPE) study [2] and that has since become a widely used intervention for cardiovascular protection. Telmisartan was better tolerated than ramipril, despite intolerance of an ACE inhibitor being screened for in the study run-in period. The parallel trial, Telmisartan Randomized AssessmeNt Study in aCE iNtolerant Subjects with Cardiovascular Disease (TRANSCEND^®^), in nearly 6,000 patients intolerant of ACE inhibitors, showed that telmisartan significantly reduced the cardiovascular composite of cardiovascular death, myocardial infarction and stroke (although not heart failure) compared with placebo, when administered on a background of otherwise optimal standard of care [3].

The ONTARGET^® ^trial established telmisartan as a proven option for the prevention of cardiovascular events in patients at high risk. Both ARBs and ACE inhibitors are currently used to slow the onset and progression of diabetic nephropathy. Recent analyses of the ONTARGET^® ^trial have provided evidence for slowing the progression of renal disease demonstrated by level of microalbuminuria in at-risk patients, including those with diabetes, being treated with telmisartan or ramipril [4,5]. These data are of particular interest given the link between renal and cardiovascular disease.

In this review, we place these results in the context of existing data on telmisartan in diabetic nephropathy, beginning with a review of renal and cardiovascular disease.

### Assessing the magnitude of renal impairment

Serum creatinine, a commonly used indicator of renal impairment, shows substantial inter-individual variability due to non-renal factors - mainly due to differences in muscle mass. To quantify accurately the degree of dysfunction, measured levels must be adjusted for the effects of age and gender; for example, to provide an estimated glomerular filtration rate (GFR). The equation developed for use in the Modification of Diet in Renal Disease (MDRD) study [6] is now widely used to measure renal function, although it is only valid for an estimated GFR < 60 mL/min (the conventional threshold for chronic kidney disease) [7]. To address the limitations of the MDRD equation, the Chronic Kidney Disease Epidemiology Collaboration equation has been developed that performs better at higher GFRs [8]. Cystatin C levels provide a more accurate measure of early stages of renal impairment, but this approach is currently not widely adopted [9].

The simplest and most widespread tool for identifying early changes in renal function is the dipstick test for albumin in early morning urine [10]. Normo-albuminuria is conventionally defined as levels < 30 mg/24 hours, microalbuminuria as 30-299 mg/24 hours, and macroalbuminuria as ≥ 300 mg/24 hours. Alternatively, albuminuria may be expressed as a urinary albumin/creatinine ratio (UACR), macroalbuminuria being ≥ 300 mg/g creatinine [11]. A new definition of total proteinuria and albuminuria has been agreed upon and demonstrated effective (Table 1) [12]. This addresses the fallacies associated with the use of the terms "microalbuminuria" and "macroalbuminuria" as being distinct entities. In reality, there is a continuous relationship between albuminuria and cardiovascular and renal outcome, and even low-level albuminuria is associated with increased cardiovascular morbidity and mortality. Although albuminuria is extensively used to identify renal impairment in patients with diabetes, it is possible to have a low estimated GFR (< 60 mL/min/1.73 m^2^) despite normoalbuminuria [13]. Such individuals, who are typically older, female, hypertensive and have cardiovascular disease, are as likely to experience a cardiovascular event as people with microalbuminuria and an estimated GFR >60 mL/min/1.73 m^2^. Both parameters should be employed to identify accurately all patients at increased risk of cardiovascular disease.

**Table 1:** New definitions of total proteinuria and albuminuria [12].

Assay	Normal	Low	High (previously termed microalbuminuria)	Very high (previously termed macroalbuminuria)
24-hour excretion (mg/24 hours)	< 10	10-29	30-300	>300
Spot UACR (mg/g)*	< 10	10-29	30-300 (males,17-250; females,25-355)	>300 (males, >250; females >355)

UACR, urinary albumin/creatine ratio.

Correspondence among terms is inexact; therefore, threshold levels are not consistent.

*For conversion of UACR expressed as mg/mmol, multiply by 0.113.

### The link between chronic kidney disease and cardiovascular disease

Chronic kidney disease is a continuum of pathology that manifests as decreased glomerular filtration and increased albuminuria. Albuminuria is an important, but somewhat overlooked, risk factor. Even small increases in albuminuria elevate the risk of end-stage renal disease (ESRD) [14], and the presence of albuminuria may be used to refine estimates of the risk of developing ESRD and cardiovascular disease compared with estimated GFR alone [15]. Once abnormal levels of albumin are detected in the urine of patients with diabetes, death from cardiovascular disease is more likely than the development of renal failure [16], although a substantial proportion of patients will still develop ESRD. A 5-year prospective study of 27,998 patients with renal impairment, of whom 15.8% were identified as having diabetes as the primary cause of renal disease, found that the incidence of death was 19.5% and that of renal replacement therapy was only 1.1% in the patients who started the study with stage 2 chronic kidney disease (estimated GFR 60-89 mL/min/1.73 m^2^) [17]. For patients with stage 3 disease (estimated GFR 30-59 mL/min/1.73 m^2^), the respective incidences were 24.3% and 1.3%. In those with stage 4 disease (estimated GFR 15-29 mL/min/1.73 m^2^), the incidence rose to 45.7% and 19.9%, respectively. The presence of diabetes at baseline resulted in a higher incidence of death for each stage of chronic kidney disease, and the relatively high incidence of death in the stage 2 cohort was attributed to patients with diabetes. Patients with diabetic nephropathy account for 27% of those undergoing dialysis [18]. Once a patient is on dialysis, the risk of dying of cardiovascular disease in an individual under 30 years old is equivalent to adding 50 years to his or her actual age [19].

Post-hoc analysis of data from the HOPE trial showed that, in addition to diabetes being associated with increased risk of renal impairment, microalbuminuria was associated with an increase in the primary cardiovascular endpoint. However, in the presence of renal impairment, the likelihood of a major cardiovascular event was similar in diabetic and non-diabetic patients; in the overall patient population, the odds ratio of 1.6 for microalbuminuria was similar to that of 1.5 for coronary artery disease [20] The Action in Diabetes and Vascular disease: PreterAx and DiamicroN-MR Controlled Evaluation (ADVANCE) showed that the reduction in the risk of a macrovascular event correlated with the reduction in a renal event [21]. Similarly, in the patients with hypertension and left-ventricular hypertrophy studied in the Losartan Intervention For Endpoint reduction in hypertension (LIFE) trial, UACRs directly correlated with the incidence of cardiovascular morbidity and mortality [22]. In the 1,063 diabetic patients in LIFE, a 10-fold increase in UACR increased the hazard ratio (HR) for the composite endpoint of cardiovascular or all-cause mortality, stroke or myocardial infarction by 39.6% and cardiovascular mortality by 46.9%. Even in patients whose values were within the normoalbuminuric range, cardiovascular risk increased according to urinary albumin levels.

### Evaluating risk in patients with renal impairment

Risk can be expressed as in absolute, individual or lifetime terms. Absolute risk is derived from epidemiological studies in large patient populations. Risk calculators provide an overall measure of the survival in the average person within a given population, and are frequently extrapolated to the general population [23]. These data are used by governments and other healthcare providers, together with evidence of the impact of specific interventions gathered from outcome trials, to determine the most cost-effective means of maintaining a large healthy population and treating those who are sick. The decisions made largely take into account the age distribution of the population; other considerations are clinical status, ethnicity and psychosocial and socioeconomic factors. However, patients at risk of cardiovascular disease have an aggregate of risk factors present at varying degrees of severity.

Individual risk estimates are based on the presence of specific modifiable and non-modifiable risk factors. Metabolic syndrome, for example, can result from the presence of various combinations of modifiable risk factors, each having an effect on the overall risk [24]. However, the use of risk calculators for patients with clearly identifiable evidence of vascular disease, such as diabetes with retinopathy or peripheral arterial obstructive disease, serves no purpose, since the need for treatment is self-evident. Another disadvantage of risk calculators is that they do not take into account early pathological changes, such as vascular calcification. Estimation of risk does not in itself provide a shortcut for therapeutic decision making. Outcomes trials can only demonstrate the benefits of treatment in a clearly defined patient population. Very high-risk patients, for example, are often excluded from clinical trials, but these are the very patients that need aggressive management. Results of subgroup analyses of large-scale trials may provide further insight into responses to treatment in patients with a specific degree of disease severity within the overall entry requirements. As a result, in selecting treatment for individual patients, physicians should be guided by the findings of outcome trials, but should be prepared to interpret the results for the specific patient under consideration.

Lifetime risk, the third of the risk formulations, gives guidance on the optimal stage for intervention. Although the data are limited, they suggest that life-long exposure to specific risk factors can drastically shorten life expectancy. Early management of these risk factors by personal and country-wide interventions can improve prognosis and extend the number of healthy life-years [25].

### Diabetic nephropathy - incidence and risk factors

Nephropathy is particularly prevalent in the diabetic patient. Based on a threshold of estimated GFR < 60 mL/min/1.73 m^2^, the prevalence among adults in the general population is 4.6% in the United States and Europe [26]. By comparison, the incidence of nephropathy in people with diabetes is 26.4%. The UK Prospective Diabetes Study (UKPDS) found high rates of renal-disease progression in type 2 diabetes, with an annual rate of transition from normoalbuminuria to microalbuminuria of 2%, from microalbuminunria to macroalbuminuria of 2.8%, and from macroalbuminuria with normal serum creatinine to serum creatinine levels >175 μmol/L of 2.3% [16]. The incidence of death increases as chronic kidney disease progresses, and the 20-year survival rate of a patient with type 2 diabetes and nephropathy is only about 50% [16].

Several pathological mechanisms link diabetes with the progression of renal disease. In the early stages of nephropathy in individuals with diabetes, a number of cellular and sub-cellular changes occur in the glomerular membrane that lead to increased permeability to proteins. Among those identified are damage to the podocytes, changes in the expression of matrix proteins, loss of selectivity and charge, as well as damage to the endothelium with consequent endothelial dysfunction [27-30]. Angiotensin II-mediated haemodynamic mechanisms increase the intra-glomerular pressure and result in the excretion of proteins [31].

The risk for diabetic nephropathy is multi-factorial and includes a family history of diabetes [32] and lifestyle factors, with a resultant high body mass index [33]. One of the more intriguing risk factors appears to be a low birth weight [34-36]. Intra-uterine programming and loss of nephrons may render the kidneys of an individual with low birth weight more susceptible to hypertension or diabetes [37]. Low birth weight is also associated with retinal arteriolar narrowing in both children and middle-aged adults [38,39].

High blood pressure is well established as an important risk factor for type 2 diabetes. In the Atherosclerosis Risk in Communities (ARIC) study, individuals with systolic blood pressure (SBP)/diastolic blood pressure (DBP) >140/90 mmHg were 2.5 times more likely to develop diabetes than their normotensive counterparts [40]. Also, high blood pressure is a strong predictor of ESRD, and the risk is amplified if blood pressure is high in patients who subsequently develop diabetes [41]. The possibility that hyperglycaemia appears to sensitize the kidneys to the harmful effect of hypertension was first mooted in 1969 [42]. In a normotensive rat model, at least, progressive proteinuria only occurs when deoxycorticosterone acetate-induced hypertension is superimposed on hyperglycemia [43].

### Effect of blood pressure control on chronic kidney disease

In people without diabetes, the benefits of lowering blood pressure on chronic kidney disease are not conclusive. The MDRD study showed that, with intensive blood pressure control that resulted in a mean arterial pressure of 91 mmHg, the decline in estimated GFR was 1.9 mL/min/year [44]. By comparison, with ordinary blood pressure control (mean arterial pressure 107 mmHg) the rate of loss was 3.4 mL/min/year. However, the difference between the two approaches to treatment was not statistically significant [44]. A 10-year follow-up, however, found that there was a 32% reduction in renal failure in patients with a mean arterial pressure of < 92 mmHg in comparison with those in whom normal blood pressure control was achieved [45].

**Figure 1:**
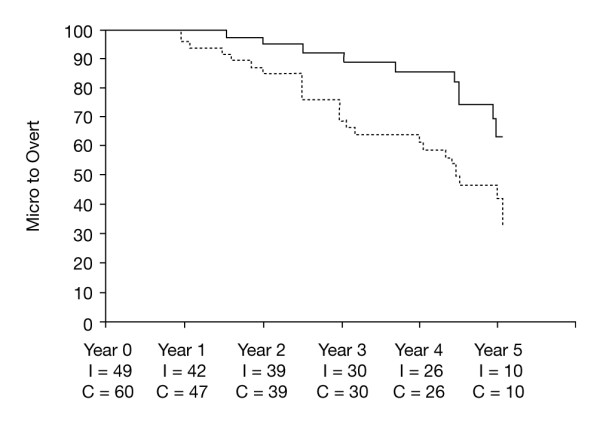
**Kaplan-Meier curve showing progression from microalbuminuria (30-300 mg/24 hours) to overt albuminuria (>300 mg/24 hours) in patients with type 2 diabetes during conventional and intensive blood pressure lowering **[46].

The evidence is more conclusive for patients with type 2 diabetes. Over a 5-year follow-up, intensive control (mean SBP/DBP 128/75 mmHg) slowed the progression from normoalbuminuria to microalbuminuria, and from microalbuminuria to macroalbuminuria, compared with conventional control, which resulted in mean SBP/DBP of 137/81 mmHg (Figure 1) [46]. The benefit of vigorous blood pressure control in patients with diabetes is supported by recently reported findings from the ADVANCE trial: renal events were least frequent if SBP was < 110 mmHg and increased log-linearly with increasing SBP [47]. Although the Irbesartan in Diabetic Nephropathy Trial (IDNT) also observed that the incidence of a renal endpoint gradually fell with the progressive lowering of SBP to < 121 mmHg after 1 year, a similar reduction in mortality was apparent only to an SBP of 121-130 mmHg, and an SBP < 121 mmHg was associated with a three-fold increase in all-cause mortality [48]. The increase in mortality may be explained by the difference in the patient populations studied: patients in IDNT were at higher cardiovascular risk because of established diabetic nephropathy and 29% had a history of cardiovascular disease [49]. However, the lower the better may not be true, as shown in the Action to Control CardiOvascular Risk in Diabetes (ACCORD) trial, in which patients with type 2 diabetes at high cardiovascular risk did not benefit from SBP lowering below 120 mmHg [50].

These findings are reflected in the lower blood pressure targets for patients with diabetes. The European Society of Hypertension/European Society of Cardiology (ESH/ESC) guidelines recognize that the target blood pressure depends on the presence of additional risk factors for cardiovascular disease and the presence of target-organ damage [51]. The presence of ≥ 3 risk factors, target-organ damage, or diabetes places a patient at high risk; thus, the recommendation is that diabetic patients with SBP/DBP >130/85 mmHg should be treated [51]. The blood pressure targets recommended in guidelines refer to office measurements. However, home or ambulatory blood pressure measurements provide a superior indication of blood pressure control [52] and may better reflect the patient's risk. One analysis has found that uncontrolled awake ambulatory blood pressure (SBP >130 mmHg) increases the risk of micro- or macroalbuminuria, even if office blood pressure was controlled [53]. Other observations in children with type 1 diabetes and elderly patients with type 2 diabetes have shown that elevated night-time blood pressure more accurately predicts albuminuria than office blood pressure [54,55]. Although association does not prove causality, use of an antihypertensive agent that provides 24-hour blood pressure control may provide better renoprotection.

### Renin-angiotensin system blockade - effects beyond blood pressure control

There is substantial evidence that drugs that act on the renin-angiotensin system (RAS) may have a beneficial effect on diabetic nephropathy, which is greater than the effects that would be expected purely from blood pressure reduction. A meta-analysis of recent studies showed that, compared with other antihypertensive classes with different mechanisms of action, treatment with ACE inhibitors or ARBs reduces new-onset diabetes by 22% in patients with or without hypertension and at high risk of developing diabetes [56]. In patients with diabetic nephropathy, for comparable levels of blood pressure achieved in IDNT using the ARB irbesartan or the calcium channel blocker amlodipine, the relative risk of developing a renal endpoint was lower with irbesartan [47]. Similarly, the Reduction of Endpoints in NIDDM with the Angiotensin II Antagonist Losartan (RENAAL) trial showed that the renal protection conferred by losartan exceeded that attributable to the small differences in blood pressure reduction compared with the control group, which received standard antihypertensive therapy, other than an ARB [57] Despite these data, one should not decry the importance of blood pressure control.

The most recent data showing the renoprotective effect of an ARB comes from the Randomized Olmesartan And Diabetes MicroAlbuminuria Protection (ROADMAP) trial [58]. This study was conducted in patients with type 2 diabetes and one additional cardiovascular risk factor (hypercholesterolaemia, low high-density lipoprotein cholesterol, hypertriglyceridaemia, obesity, large waist circumference, hypertension and/or a smoker) and who were normoalbuminuric. After adjustment for blood pressure differences, olmesartan reduced the reduction of risk of onset of persistent microalbuminuria (primary endpoint) by 17% compared with placebo, with only borderline statistical significance.

There may, however, be a distinction between renoprotection afforded by RAS blockade in patients with type 1 and type 2 diabetes based on the findings of recent studies. An evaluation of the 3,326 with type 1 and the 1,905 with type 2 diabetes, most of whom were normotensive and all were normoalbuminuric at baseline, included in the DIabetic REtinopathy Candesartan Trials (DIRECT) showed that candesartan 32 mg did not prevent microalbuminuria [59]. Another study conducted exclusively in patients with type 1 diabetes, who were both normotensive and normalbuminuric on enrolment, found that neither losartan nor enalapril slow nephropathy progression based on the change in the fraction of glomerular volume occupied by mesangium in kidney-biopsy specimens [60].

### Reducing albuminuria for renal and cardiovascular protection

Raised levels of albuminuria are associated with an increased risk of cardiovascular events. For example, in the placebo group of the Prevention of Renal and Vascular Endstage Disease Intervention Trial (PREVEND IT), the incidence of cardiovascular events was higher if albuminuria was >50 mg/24 hours than if it was ≤ 50 mg/24 hours [61]. Furthermore, reducing albuminuria lowers cardiovascular risk. In the RENAAL trial, greater reduction in albuminuria in patients with type 2 diabetes conferred greater renal protection [62]. Further analysis of the RENAAL data, which stratified patients according to the reduction in albuminuria after 6 months' treatment (0%, >0-< 30%, and >30%), showed the incidences of a cardiovascular event and of heart failure over the 48 months of observation were lowest in the patients achieving the greatest reduction [63].

### Telmisartan in diabetic nephropathy

The efficacy of telmisartan in diabetic patients with varying degrees of renal dysfunction, ranging from initial endothelial dysfunction to overt nephropathy, has been investigated in a total of five studies comprising the Programme of Research tO show Telmisartan End-organ proteCTION (PROTECTION^®^) [64].

The Telmisartan versus Ramipril in renal ENdothelium DYsfunction (TRENDY^®^) study showed that treatment with telmisartan or ramipril for 9 weeks significantly improved (p < 0.001) the response of the renal vasculature to nitric oxide, an indicator of basal nitric oxide activity and thereby of endothelial function of the renal vasculature [65]. The magnitude of the effect of telmisartan appeared somewhat greater than that of ramipril; this may explain why only temisartan improved resting renal plasma flow.

The INcipieNt to Overt: Angiotensin II receptor blocker, telmisartan, Investigation On type 2 diabetic Nephropathy (INNOVATION^®^) study, which was conducted exclusively in Japanese patients with microalbuminuria, assessed the transition to overt nephropathy when treated with telmisartan 40 or 80 mg or with placebo for up to 2 years [66]. Rates of transition were 16.7% with telmisartan 80 mg, 22.6% with telmisartan 40 mg, and 49.9% with placebo. The renoprotective effect of telmisartan was similar in the normotensive patient population and was again dose-dependent.

The Diabetics Exposed to Telmisartan And enalaprIL (DETAIL^®^) study compared the effects of the two drugs on the decline in GFR over 5 years in patients with type 2 diabetes and early nephropathy [67]. The DETAIL^® ^study used a direct, iohexol-based, measure of GFR - rather than an indirect estimation from serum creatinine levels. Both drugs provided similar renal protection. Comparison between the shorter-term ARB studies, showed that the annual decline in GFR was lowest with telmisartan [68], but the cumulative benefit due to the longer duration of the DETAIL^® ^study cannot be discounted, but it may be that the full renal protective effect of other ARBs is not achieved within the short-term follow-up [49,57,69].

**Figure 2:**
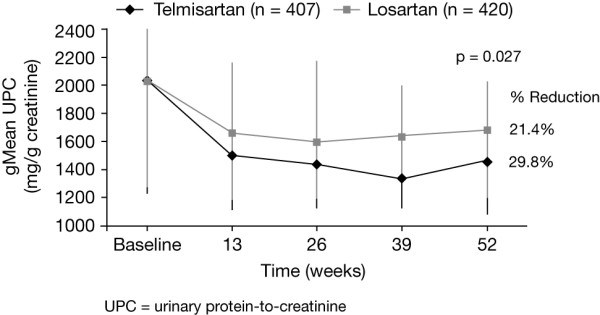
**Effects of treatment with telmisartan 80 mg or losartan 100 mg on urinary protein/creatinine ratio (UPCR) in patients with type 2 diabetes and overt nephropathy (morning spot UPCR ≥ 700 mg/g creatinine) **[70].

A prospective 1-year trial to compare Micardis^® ^versus losArtan in hypertensive type 2 DiabEtic patients with Overt nephropathy (AMADEO™) suggests that renoprotection provided by different ARBs is not the same when given at doses licensed for the treatment of hypertension [70]. The patients studied all had urinary albumin levels >900 mg/24 hours and elevated serum creatinine (97-265 μmol/L) in women and 115-285 μmol/L in men). When given for 1 year, telmisartan produced a 29.8% reduction in UACR, whereas that with losartan was only 21.4% (p = 0.027) (figure 2) [70]. This represents an approximately 30% difference in reduction of proteinuria with telmisartan 80 mg versus losartan 100 mg in favour of telmisartan. The sister trial, A trial to inVestigate the efficacy of telmIsartan versus VALsartan in hypertensive type 2 DIabetic patients with overt nephropathy (VIVALDI^®^) showed that telmisartan and valsartan provided equivalent reductions in proteinuria [71].

### Renoprotection in The ONTARGET^® ^Trial Programme

Results from TRANSCEND^® ^assessed the changes in albuminuria from baseline to after 2 years of treatment and at the end of the study [4]. The changes in albuminuria were significantly smaller with telmisartan than in the placebo plus best standard of care group. It should be noted that these data are based on observations in the overall patient population of 5,926, approximately 36% of whom had diabetes.

**Figure 3:**
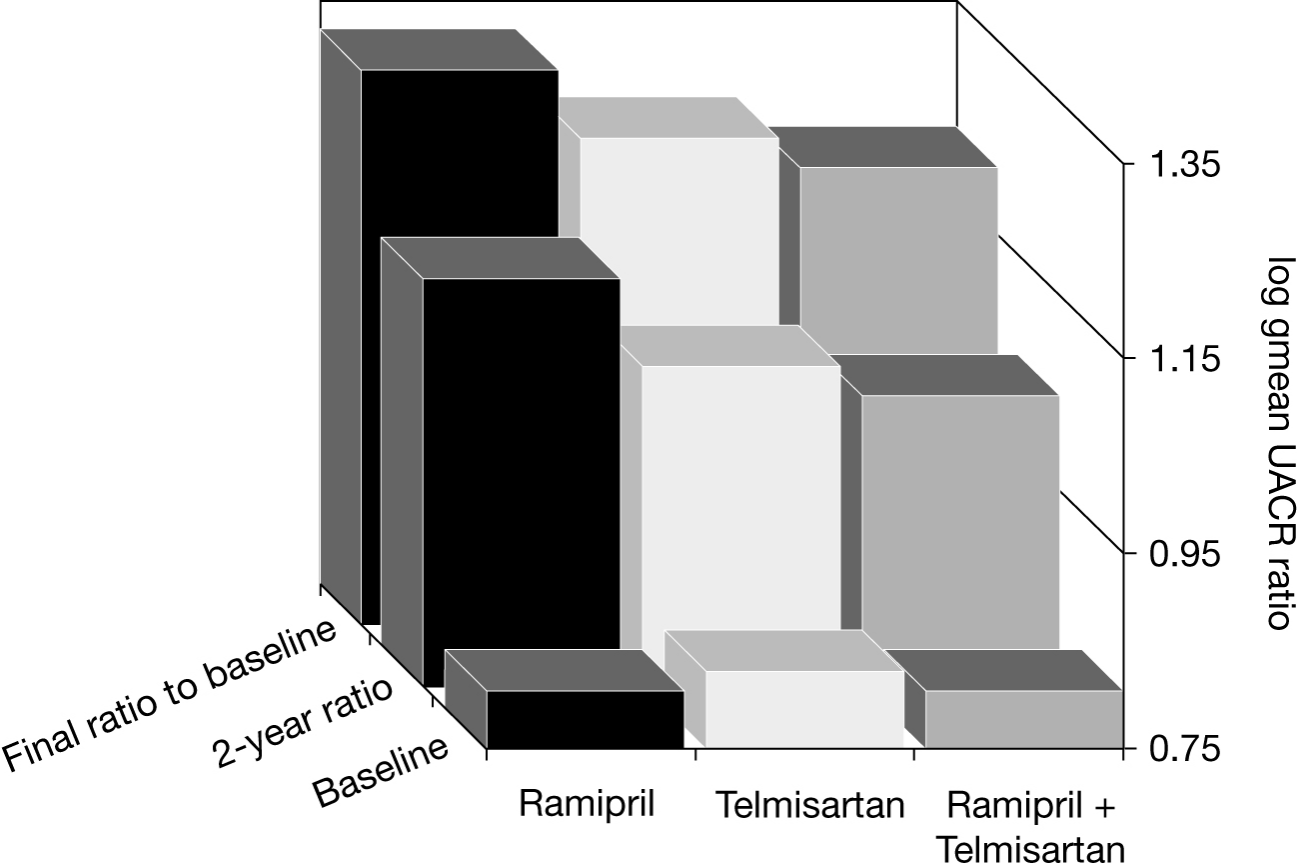
**Ratio of change in log gmean urinary albumin/creatinine ratio (UACR) after 2 years' treatment and final ratio in patients in ONTARGET^® ^treated with ramipril, telmisartan or telmisartan plus ramipril **[5].

ONTARGET^®^, the larger of the two studies with in the trial programme, demonstrated that telmisartan was more effective than ramipril in slowing down the change in albuminuria in a population of 25,620 high-risk patients and that the effect was similar to the one observed for the combination of ramipril and telmisartan [5]. The difference between treatments was apparent after 2 years, and differences between telmisartan and ramipril, and between telmisartan plus ramipril and ramipril alone, were significant at the end of the 5-year study (both p < 0.001) (Figure 3) [5]. To date, data on the renoprotection in the subgroup of 9,612 patients with diabetes are not available.

Although the combination of telmisartan plus ramipril provided superior control of albuminuria, the incidence of the primary renal endpoint for ONTARGET^® ^- a composite of dialysis (either acute or chronic), doubling of serum creatinine and death, which is the same as used in IDNT [49] - was significantly higher for telmisartan plus ramipril versus ramipril alone (HR 1.08 [95% confidence interval (CI) 1.00, 1.16]) [5]. The most frequent endpoint within the composite was all-cause mortality. If one differentiates between patients who underwent acute dialysis (i.e. < 2 months' duration) and chronic dialysis (an indicator of end-stage renal disease), the incidence of chronic dialysis was no different in the telmisartan and the telmisartan plus ramipril groups. Therefore, the driver for the difference in the primary renal endpoint was acute dialysis, which was twice as frequent in the combination of telmisartan and ramipril group compared with the ramipril monotherapy group (HR 2.19 [95% CI 1.13, 4.22], p = 0.024). Kaplan-Meier plots for dialysis reveal that the incidence of dialysis increased in the telmisartan plus ramipril group relative to the other two groups in the first 2 years of the study; subsequently, the differences diminished [5].

The decline in estimated GFR in ONTARGET^® ^has been critically examined. Over 5 years, the loss in estimated GFR was 5.94, 3.96 and 2.66 mL/min in the telmisartan plus ramipril, telmisartan and ramipril groups, respectively. The greatest loss in estimated GFR was observed in the first 6 weeks of therapy and was greatest in the patients treated with telmisartan plus ramipril. High-risk patients with cardiovascular disease are also likely to have intraglomerular vascular disease, and GFR is maintained in these patients because of the effect of angiotensin II. Hence, blockade of the RAS is expected to generate a short-term loss of GFR. The more effective the blockade, the greater the loss in GFR, which can precipitate acute renal failure and the need for short-term dialysis - which may in turn explain the high need for acute dialysis in the combination treatment arm. This decline is likely to be exacerbated if there are challenges to renal autoregulation by virtue of vascular pathology - as was likely in the ONTARGET^® ^patient population. If the annual loss of estimated GFR recorded in ONTARGET^® ^is compared with those observed in IDNT [49], IRbesartan in patients with type 2 diabetes and MicroAlbuminuria (IRMA-2) [67] and RENAAL [57], the decline in estimated GFR over 5 years was actually quite acceptable with all three treatments, comparing favourably with that observed in a general population aged between 65 and 85 years [72].

Of important note is the increase in adverse events in the combined telmisartan and ramipril arm of the study with no added benefit [1]. This increased adverse event rate with ARB + ACE inhibitor with no efficacy benefit was also seen in the VALsartan In Acute myocardial iNfarcTion (VALIANT) trial with valsartan and captopril [73]. For this reason, the combination of ARB and ACE inhibitor is not advised.

## Conclusions

An essential component of the management of the diabetic patient, especially those with other risk factors such as nephropathy, is the control of blood pressure to prevent cardiovascular events and premature death. Blood pressure targets are in most cases not being met [74]. A multifactorial approach is needed for the management of risk factors (i.e. total cholesterol, hyperglycaemia and anaemia, as well as blood pressure) in patients with diabetes. However, management of risk is lamentably poor - even by diabetologists and nephrologists [75].

Albuminuria provides a simple means of identifying the onset of kidney and vascular disease and charts the progression of chronic disease in patients with type 2 diabetes. As well as being a risk factor for ESRD, albuminuria is a prognostic factor for cardiovascular morbidity and mortality. Clinical studies in patients with type 2 diabetes show that the use of ARBs effectively protects the renal function, the effect being not exclusively attributed to blood pressure control. Post-hoc analysis of studies evaluating ARBs suggests that this approach to treatment also reaps cardiovascular benefit. Telmisartan provides superior reductions of proteinuria, compared with losartan, and is effective in reducing renal endpoints. Coupled with evidence of cardiovascular protection in ONTARGET^®^, this reinforces the potential for telmisartan as an alternative to ramipril to reduce the progression of diabetic nephropathy and to reduce cardiovascular risk in a high-risk population.

## Competing interests

ER is a member of an advisory board for Boehringer Ingelheim and is on the steering committee for the VITAL study (sponsored by Abbott) as well as the ROADMAP study (sponsored by DaiichiSankyo).

RS has received consulting and lecture fees and research grants from Boehringer Ingelheim and from other companies manufacturing angiotensin II receptor blockers.

CP has received speaker fees, advisory board payments and been provided travel to scientific meetings by various pharmaceutical companies. Boehringher ingelheim has funded an investigator initiated study in CP's laboratory in an unrelated field of research.

## Authors' contributions

ER, RS and CP were jointly responsible for the conception and content. ER, RS and CP reviewed the draft and approved the final copy.
